# An investigation of the neuroprotective effects of Curcumin in a model of Homocysteine - induced oxidative stress in the rat's brain

**Published:** 2010

**Authors:** A. Ataie, M. Sabetkasaei, A. Haghparast, A. Hajizadeh Moghaddam, R. Ataie, Sh. Nasiraei Moghaddam

**Affiliations:** 1 Neuroscience Research Center, M.C.; 2 Departments of Pharmacology, Shahid Beheshti University, M.C. Tehran; 3 Departments of Biology, Mazandaran University, Babolsar; 4 Departments of Pharmacology, Tehran University, of Medical Science, Tehran, Iran

**Keywords:** Curcumin, Homocysteine, Lipid peroxidation, Oxidative stress

## Abstract

**Background and the purpose of the study:**

Aging is the major risk factor for neurodegenerative diseases and oxidative stress is involved in the pathophysiology of them. Oxidative stress can induce neuronal damages and modulate intracellular signaling, ultimately leading to neuronal death by apoptosis or necrosis. In this study, the possible antioxidant and neuroprotective properties of the natural polyphenolic antioxidant compound, curcumin against homocysteine (Hcy) neurotoxicity was investigated.

**Methods:**

Curcumin (5, 15, 45 mg/kg) was injected intraperitonealy (i.p.) once daily for a period of 10 days beginning 5 days prior to Hcy (0.2 µmol/µl) intracerebroventricular (i.c.v) injection in rats. Biochemical and behavioral studies, including passive avoidance learning and locomotor activity tests were studied 24 hrs after the last curcumin or its vehicle injection. The cell density of hippocampus layers and apoptosis in rats’ hippocampi by immunohistochical methods were also studied.

**Results and major conclusion:**

Results indicated that Hcy could induce lipid peroxidation and increase Malondialdehyde (MDA) and Super Oxide Anion (SOA) levels in rat's brain. Additionally, Hcy impaired memory retention in passive avoidance learning test. However, curcumin decreased MDA and SOA levels significantly and improved learning and memory in rats. On the other hand Hcy could induce cell death and apoptosis in rats’ hippocampi which was inhibited by curcumin. These results suggest that Hcy may induce lipid peroxidation in rat's brain. and polyphenol treatment (curcumin) improves learning and memory deficits by protecting the nervous system against Oxidative stress.

## INTRODUCTION

Homocysteine (Hcy); a sulfur containing amino acid derived from the metabolism of methionine, is an independent risk factor for cardiovascular disease (1). The thiol group of Hcy is readily oxidized in plasma and culture medium, resulting in the generation of reactive oxygen species (ROS). Moreover, Hcy has the ability to inhibit the expression of antioxidant enzymes such as glutathione peroxidase (GSH-Px), and superoxide dismutase (SOD) (2, 3). Hcy is an excitatory amino acid, which markedly enhances the vulnerability of neuronal cells to excitotoxic and oxidative injury (2). An elevated plasma level of Hcy (more than 14 µM) is termed Hyperhomocysteinemia (HHCY) (4). Hcy is recognized as an independent risk factor for myocardial infarction, coronary artery disease, strokes, genetic disorders, Alzheimer's diseases (AD) and cognitive impairment (5). Recently, it has been suggested that chronic administration of Hcy to rats affected both long- and short-term memory in the Morris water maze task (6). Additionally, in another study it was found that in AD patients, high Hcy plasma levels favored neurodegeneration (7).

Polyphenols are natural substances that are present in plants, fruits and vegetables including olive oil, red wine and tea (8). The yellow pigment extracted from the rhizome of *Curcuma longa*, “*curcumin”* a polyphenolic non-flavonon compound is the pharmacologically active substance of turmeric (9). Curcumin is nontoxic and has antioxidant, anti-inflammatory and anti-proliferative activities. Curcumin shows antioxidant activity equivalent to vitamins C and E (10). Many studies have shown that curcumin is a potent scavenger of reactive oxygen species, which cause oxidative damage; and improves learning and memory (11). Studies of animal models have shown that curcumin inhibits lipid peroxidation and protects kidney cells, vascular endothelial cells, rat myocardium and collagen from oxidative damage (10). Vajragupta et al. showed that three Manganese complexes of curcumin exhibited a great capacity to protect brain lipids against peroxidation, enhance SOD activity and compared to other compound, showed highest inhibitory activity against H_2_ O_2_ -induced cell damage (12). Therefore, the present study was designed to investigate the neuroprotective effect of curcumin against Hcy toxicity by behavioral studies, as well as biochemical analysis to arrive at a conclusion.

## MATERIAL AND METHODS

### 

#### Drugs and Biochemical reagents

D-L-Homocysteine (Hcy), curcumin, butylhldroxy- toluene (BHT), 2-thiobarbituric acid (TBA), 1.1.3.3 tetramethoxypropan (99%), nitro blue diformazan (NBD), nitro blue tetrazolium (NBT), trichloroacetic acid (TCA), butanol and ethyl oleate, hematoxylin & eosin, DAB, xylene, caspase 3 antibody, envision antibody, diamino banzydil, hydrogen peroxide were purchased from Sigma- Aldrich and Cell Signaling company, Germany.

#### Animals

Adult male Wister rats which were purchased from Pasteur institute (Tehran, Iran), and weighing between 250–300g were used in this study. The animals were housed at 22°C in a controlled environment with a 12:12 hrs light: dark cycle and had access to standard laboratory food and water ad libitum. All experiments were carried out in accordance with the guide for the care and use of laboratory animals (National Institutes of Health Publication No. 80–23, revised 1996) and were approved by the research and ethics committee of the Shahid Beheshti University, M.C.

#### Drugs- preparation and administration

**Figure 1:**
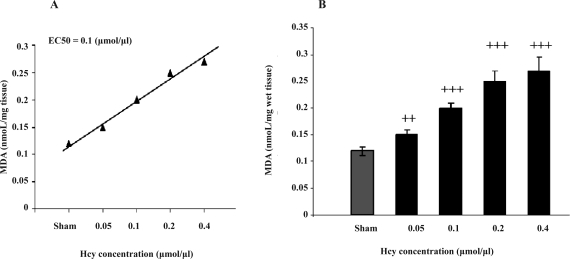
(A) Regression line and EC50 of Hcy effect on MDA concentration (B) Hcy dose-response diagram on lipid peroxidation biomarker (MDA concentration) in rats’ brain. Each point is the Mean±SEM (n=8).++ *p*<0.01, +++ *p*<0.001 difference from sham group

Hcy powder was dissolved in hydrochloric acid (1 M) and diluted with Phosphate Buffered Saline (PBS) (13). The PH of the solution was regulated at 7.4 by addition of 0.1N NaOH. Solutions of Hcy were prepared freshly at concentration of 0.05, 0.1, 0.2 and 0.4 M. Hcy effective dose (0.2 µmol/µl) was calculated by drawing a dose-response diagram (Figure 1).

The yellow powder of curcumin was dissolved in absolute ethanol and stored as stock solution (1%) at-20°C. For injection, it was diluted with ethyl oleate (vehicle) and administrated at low, middle and high doses (5, 15 and 45 mg/kg) i.p. in rats. Curcumin dosages were selected on the basis of earlier reports, which have demonstrated its antioxidant effects (15, 16). Six groups with eight animals in each group were employed in this study. Control group did not receive any injection. In the *sham* group, vehicle of Hcy (PBS) was injected (i.c.v) and vehicle of curcumin (ethyl oleate) was injected (i.p.) for 10 days beginning 5 days prior to PBS injection. In the Hcy group, Hcy (0.2 µmol/µl) (i.c.v) and the vehicle of curcumin (ethyl oleate) were injected (i.p.) for 10 days beginning 5 days prior to Hcy injection. In curcumin groups, curcumin (5, 15 and 45 mg/kg) was injected (i.p.) once daily for a period of 10 days beginning 5 days prior to Hcy (0.2 µmol/µl) i.c.v injection in rats. Biochemical and behavioral analyses were performed 24 hrs after the last injection of curcumin or its vehicle in the experimental groups.

#### Intracerebroventricular injection

The rats were anaesthetized with ketamin/xylosin (10 mg/kg) and placed in a stereotaxic frame. The rat skull was orientated according to Paxinos and Watson stereotaxic atlas (17). After a sagittal incision, the bregma suture was located and holes were drilled with an electrical drill at the following co-ordinates; 0.8 mm posterior to bregma, 1.5 mm lateral to the sagittal suture and 3.7 mm ventral. Care was taken not to damage the meninges. A Hamilton syringe with a cannula of diameter of 0.3 mm was used to inject 1µl of solutions of Hcy (0.05, 0.1, 0.2, 0.4 M) or vehicle (PBS). The injection was carried out in the brain ventricle at a rate of 1µl per 2 minutes. The cannula was left *in situ* for a further 5 min following Hcy injection to allow passive diffusion from the cannula tip and to minimize spread into the injection tract. The cannula was then slowly removed from the scalp, which was then closed with sutures. Five days after Hcy injection, rats were sacrificed by neck fracture and decapitated. The brain was removed carefully, inserted in PBS solution (0.1M) immediately and stored at -70° C for use in biochemical analysis. At the end of the study, 1µl of methylene blue solution (1%) was injected (i.c.v), to verify the site of injection (Figure 5).

#### Biochemical analysis of the brain homogenate

On the day of experiment, rat brain was weighed (estimate 1.5 g), and homogenized (10% w/v) in 0.1 M PBS with Polytron homogenizer at pH of 7.4. Homogenates were used immediately for detection of the biomarkers for lipid peroxidation, malondialdehyde (MDA) and superoxide anion (SOA). Lipid peroxidation was determined according to the modified method (18).Whole Brain homogenates (1 ml) were incubated at 37°C in an oscillating water bath for one hour. At the end of the incubation period, 0.5 ml of BHT (0.5 mg/ml in absolute ethanol) and 1 ml of TCA (25%) were added. The tubes were sealed and heated for 10 minutes in a boiling water bath to release MDA (the end product of lipid peroxidation) from proteins. To avoid adsorption of MDA to insoluble proteins, the samples were cooled to 4°C and centrifuged at 2000 x g for 20 minutes. Following centrifugation, 2 ml of the protein free supernatant was removed from each tube and 0.5 ml of TBA (0.33%) was added to this fraction. All tubes were heated for one hour at 95°C in a water bath. After cooling, the TBA-MDA complexes were extracted with 2 ml of butanol.

The light absorbance was read at 532 nm on a spectrophotometer and MDA levels were determined from standard curve that was generated from 1,1,1,3 Tetramethoxypropan. The results are represented as *n moles/mg tissue*.

For detection of SOA, the assay procedure was a modification of the method described by Das et al. (19). Brain homogenate (1 ml) was incubated with 0.4 ml of NBT (0.1%) in an oscillating water bath for one hour at 37 °C. Termination of the assay and extraction of the reduced NBT was carried out by centrifuging the samples for 10 min at 2000 x g, followed by re- suspension of the pellets with 2 ml of glacial acetic acid. The absorbance was measured at 560 nm on a spectrophotometer and converted to micromoles of Diformazan using a standard curve generated from NBD. The results are expressed as *µ moles/mg tissue*.

#### Hippocampus histopathological analysis

Because of the important role of hippocampus in the memory, its histopathological was investigated. So removed rat's hippocampal tissues were fixed in 10% neutral buffered formaldehyde for 24 hours, embedded in paraffin and cut into 3–4 µm thick sections by a microtome (Leica SM2000R, Germany). The tissue sections were de-paraffinised in xylene. The slides were stained with Hematoxylin and E o s i n (H&E) according to the procedure of Wilson et al. (35) and viewed under a light microscope (Labomed, USA) for the structure and morphology of cells. Microscopic images obtained by a CCD camera and Digipro software. The cells also were counted in different regions of hippocampus (CA1, CA2, CA3 and DG) by the grade of light microscope. The results are represented a cell count per mm2 tissue. Also some of slides were investigated by Immunohistochemical methods. In these processes, the primary and secondary antibodies were used and Caspase 3 (an apoptotic cytoplasmic protein) was detected as brown color after making complex with DAB (Diamino Banzydil).

#### Behavioral study

The effects of Hcy alone or in combination with curcumin on the rats’ behavior were studied by passive avoidance learning task. Locomotor activity was also studied by *Open field* apparatus. All studies were begun 24 hrs after the last curcumin or its vehicle injection in the fifth day following Hcy (i.p.) injection.

#### Passive avoidance learning apparatus

The apparatus (shuttle box) consisted of equal sized light and dark compartments (30×20×30 cm), separated by a guillotine door (7×9 cm) that could be raised to 10 cm. A 40 W lamp was fixed 30 cm above the floor in the center of the light compartment. The floor was made from stainless steel (2.5 mm in diameter) and connected to a shock stimulator. A single electrical shock (50 Hz, 1.5 sec, 1.5–2 mA intensity) were delivered to the grid floor of the dark compartment by a stimulator.

#### Training

Five days after Hcy (i.c.v) injection, the rats were individually allowed to habituate to the apparatus one hour prior to testing. All behavior tests were performed at 9:30 – 10:30 AM in a darkened, light and sound attenuated and ventilated testing room at 20–25°C. Each animal was placed in the light compartment, and after 5 sec, the door was opened, and animals were allowed to enter a dark place. The habituation trial was repeated after 30 minutes followed by the acquisition trial after an additional 30 minutes. Thus, every animal was placed in the light compartment and the latency time that elapsed before each animal had all four feet inside the dark compartment was measured in seconds and termed “Initial Latency” time (IL). Immediately after the rat entered the dark chamber with all the four feet inside, the door was closed and an electrical foot shock (1.5–2 mA, 50 Hz, 1.5 sec) was delivered. After 20 sec, the rats were removed from the dark chamber and returned to their own cages. Rats that had an IL time of more than 100 sec were excluded from further experiments. Any rat that was excluded in initial latency test, replaced with the new rat so the number of rats in each groups were kept fixed at 8. Two minutes later, the rats were re-tested in the same way. If the rats did not enter the dark compartment during 120 sec, successful acquisition of passive avoidance response would be recorded (20).

#### Retention test

Twenty-four hrs after training, the retention test was performed. Each rat was placed in the light compartment for 5 sec, the door was opened and the step through latency time was measured and termed the “Retention Latency” time (RL); but foot shock was not delivered. The test was concluded when the animal entered the dark compartment or remained in the light site for 600 sec as an upper cut of time (20).

#### Open field test

The general stimulant or depression activity of a CNS-active drug may affect the response of an animal on behavioral paradigm. Therefore, the effect of curcumin and Hcy on spontaneous locomotor activity in open field were also studied. Spontaneous locomotor activities were measured on days of 5, 6 after Hcy (i.c.v) injection. In these days initial and retention latency times were also measured. Each animal was observed over a period of 5 min in a square open arena (30×30 cm) equipped with an infrared light-sensitive photocell using a digital photoactometer and recorded by a video path analyzer. Measurements were expressed as locomotion, rest; distances traveled and speed of the animal. (20, 21).

#### Statistical analyses

The data of behavioral, biochemical and histopathological studies were analyzed using one- way analysis of variance (ANOVA). If the F values were significant, the student *Newman- Keuls* test was used to compare the experimental and control groups. *P-values* less than 0.05 were considered to be statistically significant.

## RESULTS

### 

#### Hcy Dose-Response diagram

Figure 1 shows Hcy dose-response diagram on rat's whole brain lipid peroxidation, dose- response regression line and EC_50_ It was found that 0.2 µmol/ µl was the maximum safe effective dose of Hcy to induce lipid peroxidation. Also EC50 was 0.1 µmol/µl. Higher concentration of Hcy (more than 0.4 µmol/µl) caused side effects, some morbidity and mortality in rats

#### Estimation of oxidative stress parameters

**Figure 2:**
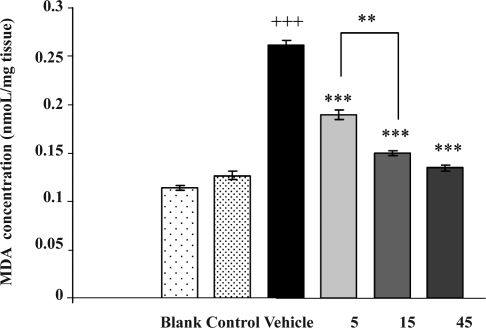
Effects of Curcumin (5, 15 and 45 mg/kg) on MDA concentration of rats’ brain in Hcy (0.2µmol/µl) treated rats. Curcumin at the dose of 45mg/kg decreased MDA concentration less than 5 mg/kg (*p*<0.01). In the Control group nothing were injected, In the Sham group, vehicles of Hcy (i.c.v) and Curcumin ( i.p.) were injected. Each point is the Mean±SEM (n=8). ** *p*<0.01, difference between two doses of curcumin, ** *p*<0.001 difference from Hcy group, +++ *p*<0.001 difference from sham group.

Figure 2 shows the effects of drugs on lipid peroxidation. One-way ANOVA indicated that MDA level in Hcy group was increased significantly as compared to the sham group [*F* (5, 42) =164.54, *p*<0.001]. Curcumin treatment (5, 15 and 45 mg/ kg; i.p.) decreased the MDA level significantly (*p<*0.001) and at 45 mg/kg decreased the MDA level less than that of 5 mg/kg (*p<*0. 01).

**Figure 3:**
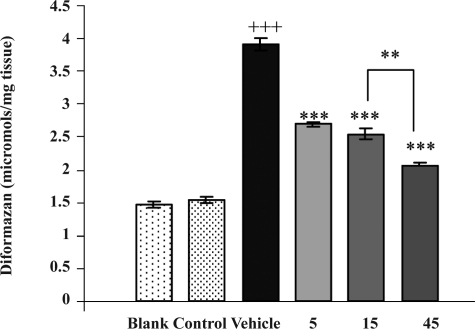
Effects of curcumin (5, 15 and 45 mg/kg) on Super oxide anion (Diformazan) concentration of rats’ brain in Hcy (0.2 µmol/µl) treated rats. Curcumin at the dose of 45mg/kg decreased SOA (Diformazan) concentration less than 5 mg/kg (*p*<0.01). In the control group nothing was injected, In the Sham group, vehicles of Hcy (i.c.v) and curcumin (i.p.) were injected. Each point is the Mean±SEM (n=8). ** *p*<0.01, difference between two doses of curcumin, *** *p*<0.001 difference from Hcy group, +++ *p*<0.001 difference from sham group

Figure 3 shows the effects of drugs on superoxide anion level (µmol/mg wet tissue) in the rats’ whole brain. One-way ANOVA indicated that SOA level in Hcy group was significantly greater than that of sham group at doses of [F (5, 42) =220.98, *p*<0.001]. Curcumin treatment with 5, 15 and 45 mg/kg led to a significant decrease in SOA level (*p<*0.001) and at 45 mg/kg decreased the SOA level less than those of 5, 15 mg/kg doses (*p<*0. 01).

#### Estimation of histopathological parameters

**Figure 4:**
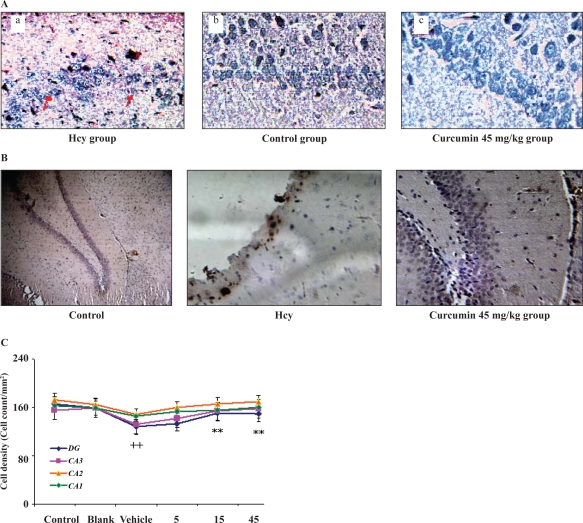
**A**: Photographs were taken from rats’ granular cells of hippocampus dentate gyrus stained with Hematoxylin and Eosin. **B:** Immunohistochemical analysis of rat's granular cells of hippocampus dentate gyrus in experimental groups. **C**: Cell density curve from different layers of rats’ hippocampus sections. Each point is the mean ±SEM. ** *p*< difference from Hcy group, ++ *p*<0.001 difference from sham group.

One-way ANOVA indicated that the cell density of Dentate Gyrus (cell count per mm^2^) in Hcy group (Figure 4a) was significantly (*p<0.001*) lower than that of the sham group (Figure 4b). However, after curcumin treatment the cell density was increased significantly in comparison with Hcy group at doses of 15 and 45 mg/kg (*p<0.01*).

Immunohistochemical study (Figure 4c) indicated that the cell layer of dentate gyrus in control group did not show any apoptotic changes. But in Hcy group the color of cytoplasm was changed to brown indicating the reaction of Caspase 3 with antibody and so the possibility of apoptosis may have occurred. After curcumin (45 mg/kg) treatment the cytoplasm brown color was disappeared. Therefore apoptosis was inhibited upon treatment with curcumin.

#### Estimation of learning and memory parameters

**Figure 5:**
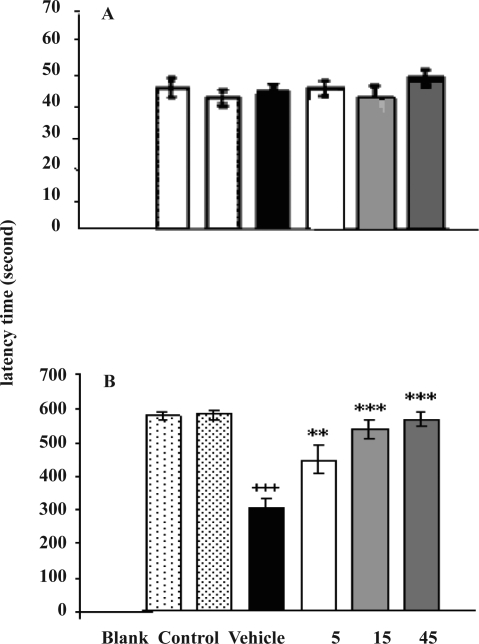
Effects of curcumin (5, 15 and 45 mg/kg) on the passive avoidance behavior after Hcy (0.2 µmol/µl) i.c.v. injection in rats. The effects of drugs on **(A)** Initial latency times (IL) before electrical shock in training process (n=8), **(B)** Retention Latency times (RL), 24 hrs after electrical shock in the retention test (n=8). In the Control group nothing was injected, In the Sham group, vehicles of Hcy (i.c.v) and Curcumin (i.p.) were injected. Each point is the Mean±SEM (n=8). *** *p*<0.001 difference from Hcy group, +++ p<0.001 difference from sham group

In this set of experiments, Panel a in Figure 5 represents initial latency times that was recorded in the training process before electrical shock and these times were naturally less than the retention latency times (RL) (panel b) that was recorded in retention test, 24 hrs after electrical shock. One-way ANOVA indicated that initial latency time (IL) did not differ between control and experimental groups (Figure 5a) while the mean RL times (Figure 5b) in Hcy group was significantly [*F* (5, 42) =18.50, *p* <0.001] less than that in sham group (*p<*0.001). However, curcumin at the doses of 5 mg/kg (*p*<0.01) and 15, 45 mg/kg (*p*<0.001) significantly reversed the Hcy-mediated decrease in step through latency time and increase in the mean of RL times (*p<*0.001).

**Figure 6:**
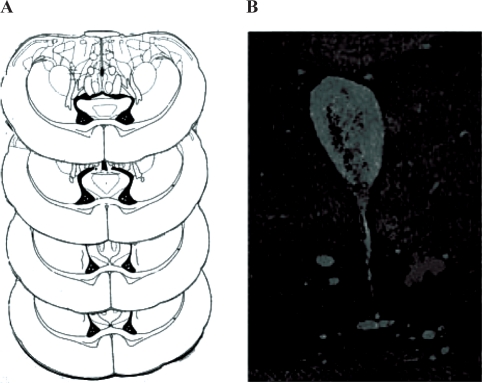
**A-** The approximate placements of injection cannulae within the cerebral ventricle are indicated by white spots. Representative sections of the ventricle (− 0.3,−0.4,−0.8, −0.92 mm from bregma) were taken from the rat brain atlas of Paxinos and Watson (1998). **B**- Histological photograph taken from rat's ventricles after methylene blue (i.c.v) injection.

#### Locomotor activity

One-way ANOVA indicated that there were not any significant differences in locomotor activities between control and experimental groups on the days of 5, 6 after Hcy (i.c.v.) injection.

## DISCUSSION

The aim of this study was to investigate and clarify the neuroprotective effects of curcumin, a polyphenolic non-enzymatic antioxidant agent, against Hcy neurotoxicity. The goal was to establish an animal model of Hcy- induced oxidative stress in the rat's brain according to previous investigations (22–24). The present study indicated that Hcy was neurotoxic for rats. Biochemical results revealed that five days after Hcy (i.c.v) injection, MDA and SOA levels were significantly increased in the whole brain tissue in comparison with the sham and control groups (Figure 2 and 3). In the present study, it was observed that Hcy overdose can be lethal for rats and some morbidity and mortality were observed after administration of 0.4 µmol/µl or higher concentrations. Furthermore, results of this study indicated that Hcy (0.2µmol/µl) could impair the memory significantly, and decreased latency time in the retention test. As it is shown, Hcy group had significantly lower latency time than other groups (Figure 5). The effects of Hcy on the memory have been investigated in some of the earlier studies. It has been found that long-term exposure of Hcy induced alteration in spatial learning, hippocampal signaling and synaptic plasticity (25).

Streck et al. (26) revealed that chronic hyper- homocysteinemia impaired memory in the rat. It was suggested that Hcy might generate reactive oxygen species (ROS), which attack the poly unsaturated fatty acids (PUFA) of neuronal cell membranes and induce lipid peroxidation in the rat brain (26). Hcy may also modulate intracellular signaling, ultimately leading to neuronal death via apoptosis or necrosis (27). ROS are highly reactive for all biological macromolecules (e.g. proteins, DNA and lipids).

PUFAs are extremely sensitive to free radicals due to their double bonds. ROS attacks on PUFAs, initiates a chain reaction called “lipid peroxidation” (28). In the course of this process, many detrimental metabolites, such as peroxides, aldehydes, ketones and alcoholes are generated. Peroxides and aldehydes, especially 4-hydroxy-nonenal (HNE) and MDA, are the key-factors for the initiation and propagation of atherosclerosis, inflammation, cancer and neurodegenerative diseases (29).

In the present study the loss of dentate gyrus cells by Hcy was observed. The role of dentate gyrus of hippocampus on memory was investigated in previous studies. Kim et al, (22) investigated the effect of treadmill exercise on memory in relation to neurogenesis and apoptosis in the hippocampal dentate gyrus of old-aged rats (36). They showed that loss of memory by aging was associated with a decrease in neurogenesis and an increase in apoptosis in the hippocampal dentate gyrus. McHugh et al. (37) showed dentate gyrus NMDA receptors mediate rapid pattern separation in the hippocampal network (37). They tested this hypothesis by generating and analyzing a mouse strain that lacks the gene encoding the essential subunit of the N-methyl-D-aspartate (NMDA) receptor NR1, specifically in dentate gyrus granule cells. The mutant mice performed normally in contextual fear conditioning, but were impaired in the ability to distinguish two similar contexts.

Curcumin treatment at low, middle and high doses (5, 15 and 45 mg/kg) inhibited lipid peroxidation significantly and decreased MDA and SOA levels in the Hcy-treated rats’ brain (Figs. 2 and 3). On the other hand, the higher dose of curcumin (45 mg/kg) decreased lipid peroxidation less than that of 5 mg/kg dose (*p*<0.01). Nonetheless, curcumin also improved Hcy-mediated memory deficits and increased latency times in the passive avoidance tests (Figure 5). According to results, curcumin has a dose-related antioxidant effects on the whole brain tissue. Curcumin by itself did not affect the learning and memory but improved these functions which were impaired by Hcy. In a previous study, Pan et al. (11) indicated that curcumin improved learning and memory in mice and investigated the neuroprotective effect of curcumin on the memory of AD mice in the step-through test (11). Curcumin reduced the number of step-through errors and prolonged step- through latency in AD mice (11). Curcumin also attenuated the neuropathological changes in the hippocampus and inhibited apoptosis (11). Lim et al. showed that curcumin reduced oxidative damage and amyloid pathology in an Alzheimer transgenic mouse (30). They also showed that low and high doses of curcumin significantly lowered oxidized proteins and interleukin-1beta, a proinflammatory cytokine, elevated in the brains of these mice (30). Kumar et al. (31) also showed that chronic treatment with curcumin (10, 20 and 50 mg/kg; per os) once daily for a period of 8 days beginning 4 days prior to 3- Nitropropionic acid (3-NP) administration, dose-dependently improved the 3-NP-induced motor and cognitive impairment (31). Biochemical analysis revealed that curcumin administration significantly attenuated 3-NP-induced oxidative stress in the brains of rats (31). Another study showed that chronic treatment with curcumin (5–50 mg/kg; per os) twice daily for a period of 25 days beginning 4 days prior to colchicines injection significantly improved the Colchicines-induced cognitive impairment. Also, chronic administration of curcumin significantly reduced the colchicines- induced elevated lipid peroxidation (32). There are multiple biological activities of curcumin (33), such as, anti-inflammatory activity via down regulation of cyclo-oxygenase 2 and nitric oxide synthase through suppression of NF-kappa B activation (34).

Results of this study suggest that the antioxidant property of curcumin may be responsible for protection against Hcy oxidative stress, possibly by increasing the endogenous defenses against oxidative stress. Results of this study suggest that curcumin may scavenge SOA from the whole brain tissues. Therefore, curcumin has protective effects against lipid peroxidation and decreased MDA and SOA formation. Also curcumin at the dose of 45mg/kg inhibited cell death (apoptosis) in hippocampus.

In conclusion, curcumin could prevent neurotoxicity of Hcy in the rat's brain. If Hyperhomocysteinemia is one of the pathological reasons for neurodegenerative disorders such as sporadic AD or Parkinson's disease, curcumin may be an effective prophylactic agent in the prevention of oxidative stress by Hcy. Furthermore, data of this study suggest that curcumin's mechanism for protection of the brain against the toxicity of Hcy may be inhibition of brain lipid peroxidation and improvement of learning and memory deficits in rats. Further studies are required to reveal the exact mechanism of Hcy in cell death process (apoptosis or necrosis) and the neuroprotective properties of curcumin must be studied in more detail.
